# Investigating the Interplay of SARS-CoV-2 RNAemia and Peripheral Inflammation in Platelet Dysfunction During Acute SARS-CoV-2 Infection

**DOI:** 10.20411/pai.v10i2.823

**Published:** 2025-07-09

**Authors:** Mariangela Scavone, Roberta Rovito, Claudia Ghali, Antonella Fioretti, Bianca Clerici, Elena Bossi, Camilla Tincati, Andrea Santoro, Elisa Borghi, Gian Marco Podda, Giulia Marchetti

**Affiliations:** 1 Division of General Medicine II, Department of Health Sciences, ASST Santi Paolo e Carlo, University of Milan, Milan, Italy; 2 Clinic of Infectious Diseases and Tropical Medicine, Department of Health Sciences, ASST Santi Paolo e Carlo, University of Milan, Italy; 3 Clinical Microbiology, Department of Health Sciences, ASST Santi Paolo e Carlo, University of Milan, Italy

**Keywords:** COVID-19, Platelets Dysfunction, SARS-CoV-2 viral RNA, Chemokines and Cytokines, Inflammation

## Abstract

**Background:**

Circulating degranulated platelets have been described during acute SARS-CoV-2 infection and associated with COVID-19 complications. This study investigated the relationship between the presence of plasma SARS-CoV-2 RNA (ie, SARS-CoV-2 RNAemia), systemic inflammation, and platelet dysfunction in a group of patients with COVID-19. Unlike our previous publication, which focused on platelet characterization, this work explores potential determinants of platelet activation, based on a distinct subset of patients with available stored samples.

**Methods:**

Patients with COVID-19 were stratified by platelet δ-granule content using the luciferin/luciferase assay into 2 groups: normal (COV_δ-norm_) and low (COV_δ-low_). Plasma SARS-CoV-2 RNAemia (RT-qPCR), cytokines, and chemokines (Cytometric Bead Array) were quantified on plasma samples. Markers of platelet activation were measured by flow cytometry in whole blood.

**Results:**

A total of 75 patients with COVID-19 were enrolled; 57 presented normal levels of platelet δ-granule content (COV_δ-norm_) and 18 had low levels of platelet δ-granules (COV_δ-low_). Groups were comparable in terms of age, sex, comorbidities, and SARS-CoV-2 RNAemia levels. Patients in the COV_δ-low_ group showed significantly higher chemokine and cytokine levels compared to those in the COV_δ-norm_ group, with strong correlations between IL-6, as well as granulocyte-macrophage colony-stimulating factor (GM-CSF), with platelet degranulation parameters. A similar trend, albeit less pronounced, was observed when patients were stratified based on their platelet activation phenotype.

**Conclusions:**

These findings suggest that peripheral inflammation, rather than SARS-CoV-2 RNAemia, is associated with platelet dysfunction during acute SARS-CoV-2 infection.

## INTRODUCTION

COVID-19 is characterized by a heterogeneous spectrum of clinical manifestations, ranging from flu-like symptoms to life-threatening multi-organ dysfunction [1]. The severe form of COVID-19, which occurs in approximately 14% of infected individuals [2], often leads to thrombotic and bleeding complications [3]. Since the beginning of the pandemic, there have been early *post-mortem* descriptions of platelet thrombi in the microcapillaries of the lung, heart, kidney, and skin of patients with COVID-19 [4]. Because of these findings, anticoagulation has been widely used in the management of inpatients with COVID-19 who are, however, also prone to bleeding [5]. One of the contributors for such a unique thrombotic and bleeding tendency might be the presence of activated and degranulated platelets in COVID-19 patients [6–9]. The mechanisms underlying the aberrant activation and degranulation of platelets in COVID-19, however, remain to be elucidated. SARS-CoV-2 RNA has been detected in various tissues during acute COVID-19, including the bloodstream [4, 10–14]. It is detectable as early as the first week of infection [15, 16] and is associated with inflammation, tissue damage, disease progression, and death [17–19]. Viral persistence may also contribute to long-COVID, although the exact mechanisms of viral dissemination remain unclear, with the bloodstream likely playing a key role [20]. SARS-CoV-2 RNA has been found in platelets of patients with COVID-19, often in the context of elevated pro-inflammatory cytokines and platelet hyperactivation [8, 21]. In some cases, the presence of replication-competent virus in platelets has been associated with fatal outcomes [22], suggesting that the virus may enter platelets during their formation in the bone marrow, similarly to other viral infections such as HIV, dengue, and influenza [23–25]. In addition, SARS-CoV-2 can interact directly with platelets [26] via the ACE2 receptor and other platelet receptors [27, 28]. During conditions such as sepsis or COVID-19, platelets are not merely innocent bystanders but key contributors of systemic inflammation [29, 30]. Unfortunately, the severe inflammation observed in COVID-19, driven by chemokines and cytokines, such as TNF, IL-6, and G-CSF [20, 31], further complicates the understanding of platelet dysfunction. Therefore, the aim of the present study was to investigate the interplay of SARS-CoV-2 RNAemia as well as peripheral inflammation with platelet dysfunction during acute COVID-19.

## METHODS

### Study Population

This study is based on a distinct subset of patients from the cohort described in our previous publication [9] with available stored plasma samples and complete data on δ-granule content and platelet activation markers. Unlike the previous analysis, which focused on platelet dysfunction per se, the current study investigates the pathophysiological relationship between SARS-CoV-2 RNAemia, systemic inflammation, and platelet dysfunction. The cohort consisted of individuals with SARS-CoV-2 infection, confirmed through RT-PCR on nasopharyngeal swab (ie, NP), enrolled between March 2021 and August 2022. Patients were hospitalized or referred to the outpatient clinic of the Department of Infectious Diseases and Tropical Medicine, University of Milan, ASST Santi Paolo e Carlo, Milan, due to their high risk of developing severe COVID-19. Patients on antiplatelet therapy or with pre-existing platelet dysfunctions were excluded from the study. The time of blood sampling since the onset of symptoms of acute SARS-CoV-2 infection was recorded for each study participant. The study was approved by the Institutional Ethics Committee (Comitato Etico ASST Santi Paolo e Carlo; 2020/ST/049, 2020/ST/049_BIS, 11/03/2020), and written informed consent was obtained from all study participants. All research was performed in accordance with the Declaration of Helsinki.

### Blood Collection

Each eligible patient underwent a single blood withdrawal for the purposes of the study. Venous blood samples were collected from the antecubital vein using a 21-gauge butterfly needle without applying a tourniquet to minimise platelet activation. The first 4 mL of blood were drawn into K2E EDTA tubes (Greiner) and analyzed for blood cell counts using a coulter haematology analyzer (Medonic M series 16). The remaining blood was anticoagulated with 109 mmol/L^-1^ trisodium citrate (9:1; vol:vol) for the assessment of platelet activation markers, such as platelet-monocyte aggregates and platelet nucleotide content, while K2E EDTA tubes were used for the quantification of plasmatic cytokines/chemokines and SARS-CoV-2 RT-qPCR analysis.

### Platelet Parameters

To evaluate platelet degranulation, intracellular levels of ADP and ATP were measured in platelet-rich plasma. These nucleotides are stored in dense granules and released upon platelet activation, leading to a marked decrease in intracellular ADP and a consequent increase in the ATP/ADP ratio. Platelet degranulation was assessed with the measurement of the content of platelet nucleotides, performed as previously described [9]. Briefly, a total of 0.5 mL of citrated platelet-rich plasma (PRP) was mixed with 0.05 mL EDTA (100 mM) and 450 µL absolute ethanol, followed by centrifugation at 16,000*g* for 45 minutes at 4 °C. The supernatant was then stored at -80 °C for further analysis. The total platelet content of ADP and ATP was measured using the firefly luciferin/luciferase method in a lumiaggregometer (Chrono-log V400) [32]. ADP and ATP levels were expressed as nmol/10^8^ platelets, and the overall ATP/ADP ratio was calculated. Cut-off values for ADP and the ATP/ADP ratio were determined based on the 10^th^ percentile (1.98 nmol/10^8^ platelets) and 90^th^ percentile (3.47 ratio), respectively, of a cohort of 36 healthy participants [9]. Platelet activation was indirectly assessed by quantifying circulating platelet-monocyte aggregates (PMAs), which form through the interaction between P-selectin on activated platelets and P-selectin glycoprotein ligand-1 on monocytes. As previously described, [9] 15 µL of citrated whole blood was stained with fluorescein isothiocyanate (FITC)-conjugated anti-human CD14 and allophycocyanin (APC)-conjugated anti-human CD41a for 20 minutes at room temperature in the dark. Samples were fixed, and erythrocytes were lysed by adding 0.5 mL of FACS Lyse Solution (BD Biosciences). PMAs were identified as double positive events for both platelet and monocyte markers (CD41a^+^ - CD14^+^). Data were analyzed using FACS Suite software (BD Biosciences), with results expressed as the percentage of positive cells. The cut-off value for PMAs was determined based on the 90^th^ percentile of a cohort of 36 healthy subjects and was set at 23.02%.

### Cytometric Bead Array

Plasmatic cytokines (IFN-α, IFN-γ, IL-2, IL-4, IL-5, IL-6, IL-9, IL-10, IL-12, IL-17A, and TNF-α) and the chemokine GM-CSF were quantified using the Human MACSPlex Cytokine 12 Kit (Miltenyi Biotec) according to the manufacturer's instructions. Briefly, thawed plasma samples were diluted 1:4 with assay diluent and incubated with MACSPlex Cytokine 12 Capture Beads for 2 hours, followed by 1-hour incubation with the MACSPlex Cytokine 12 Detection reagent. Samples were resuspended in 0.3 mL of assay buffer, acquired on a FACS Verse Cytometer (BD Biosciences), and analyzed with FlowLogic v8 software (Inivai Technologies). A total of 7 individuals sampled before the pandemic era were included as controls. Samples were acquired with FACS Verse Cytometer (BD Biosciences), and data were analyzed with FlowLogic 7.3.

### Plasmatic SARS-CoV-2 RT-qPCR

Viral RNA was extracted from 140 µL of thawed plasma by using the QIAamp Viral RNA Mini Kit (QIAGEN) and quantified by real-time PCR using the CDC 2019-nCoV_N1 primers and probe set (Centers for Disease Control and Prevention, Update June 2020) and the TaqPath™ 1-Step RT-qPCR Master Mix CG (ThermoFisher), which quantify the Nucleocapsid gene of SARS-CoV-2. The 2019-nCoV_N Positive Control plasmid (Integrated DNA Technologies, Inc.) was used for absolute quantification, while a non-template condition was used as negative control. The RPP30 quantification was employed for RNA extraction quality. The assay was performed in duplicate.

### Statistical Analysis

Eligible COVID-19 patients were categorized in 2 groups based on their platelet degranulation status, which was defined by the cut-off values of ADP and ATP/ADP (ADP content below 1.98 nmol/10^8^ platelets and ATP/ADP ratio above 3.47, respectively). Continuous variables were compared using the Mann-Whitney U test or the unpaired *t*-test, according to data distribution, which was assessed using the D'Agostino Pearson test. Categorical variables were compared using Fisher's exact test. Correlations between SARS-CoV-2 RNAemia or peripheral cytokine/chemokine levels and platelet markers were assessed using Pearson's or Spearman's correlation tests, according to data distribution. Data were analyzed and visualised using GraphPad Prism version 9.0 (GraphPad Software). Two-tailed *P*-values <0.05 were considered statistically significant.

## RESULTS

### Characteristics of Study Participants

A total of 75 patients with COVID-19 were included in the present study. The demographic and clinical characteristics of the study population are summarized in Table 1. Fifty-seven patients with COVID-19 were classified as having non-degranulated platelets (COV_δ-norm_) and 18 patients with COVID-19 as having degranulated platelets (COV_δ-low_). Age, sex, and comorbidities were comparable between the groups, with a median age of 66 years (IQR: 51-75) for COV_δ-norm_ patients and 56 years (IQR: 38-75) for COV_δ-low_ patients (*P*= 0.295) and a predominance of men in both groups (53% and 72%, respectively, *P*=0.143) (Table 1). Furthermore, patients in the COV_δ-low_ group exhibited significantly higher neutrophil/lymphocyte ratios and significantly higher levels of C-reactive protein, D-dimer, and lactate dehydrogenase compared to the COV_δ-norm_ group (Table 1). Significantly more patients in the COV_δ-low_ group received low-molecular-weight heparin, were hospitalized, or died within 30 days, and none of them received SARS-CoV-2 antivirals (Table 1).

**Table 1.** Demographic and Clinical Characteristics of the Study Patients

	COV_δ-norm_ (n=57)	COV_δ-low_ (n=18)	*P*-value
**Clinical characteristics**			
**Age (years)**, median (IQR)	66 (51-75)	56 (38-75)	0.295
**Sex**, male, n (%)	30 (53)	13 (72)	0.143
**COVID-19 vaccine**, n (%)	38 (67)	8 (44)	0.092
**Platelet parameters**			
Platelet δ-granules content:			
ADP (nmol/10^8^ plts), median (IQR)	2.99 (2.34-3.54)	1.61 (1.26-1.81)	**<0.001**
ATP/ADP ratio, median (IQR)	2.91 (2.41-3.30)	4.66 (4.26-5.50)	**<0.001**
Markers of platelet activation:			
Platelet-monocytes aggregates, (%), median (IQR)	21.9 (15.7-37.9)	39.3 (18.9-67.8)	**0.029**
**Comorbidities**, n (%)			
Obesity	12 (21)	5 (28)	0.552
Hypertension	24 (42)	8 (44)	0.861
Diabetes	4 (7)	2 (11)	0.579
Cardiovascular disease	11 (19)	4 (22)	0.787
Chronic pulmonary disease	10 (18)	1 (6)	0.210
Chronic kidney disease	4 (7)	2 (11)	0.577
Mild liver disease	2 (4)	0 (0)	0.421
Neurologic disease	4 (7)	1 (6)	0.828
Psychiatric disorder	3 (5)	0 (0)	0.321
Autoimmune disease	4 (7)	1 (6)	0.828
Transplant	2 (4)	0 (0)	0.421
Hematologic disorder	4 (7)	2 (11)	0.577
**Laboratory values**, median (IQR)			
Haemoglobin (g/dL)	12.3 (11.6-14.0)	12.7 (11.8-13.9)	0.803
Haematocrit (%)	37.7 (35.2-43.1)	39.0 (36.5-41.6)	0.784
Platelets (x10^3^/mmc)	209 (172-299)	239 (162-305)	0.597
WBC count (x10^3^/mmc)	5.9 (3.7-7.9)	6.0 (5.3-8.5)	0.586
Neutrophils (x10^3^/mmc)	3.4 (2.1-5.9)	4.8 (4.1-7.1)	0.071
Lymphocytes (x10^3^/mmc)	1.1 (0.8-1.8)	0.8 (0.5-1.1)	**0.015**
NL ratio	2.8 (1.2-6.4)	6.7 (3.6-11.4)	**0.003**
C-reactive protein (mg/L) [n/N]	23.4 (8.6-52.2)[54/57]	47 (23.0-77.3) [18/18]	**0.040**
Prothrombin time (INR) [n/N]	1.1 (1.0-1.2) [55/57]	(1.0-1.3) [18/18]	0.837
D-dimer (ng/mL) [n/N]	237 (0-332.8) [34/57]	341 (286-487) [15/18]	**0.005**
Lactate dehydrogenase (U/L) [n/N]	221 (192-290) [53/57]	301 (227-462) [18/18]	**0.033**
PF ratio at admission [n/N]	278 (208-311) [32/57]	276 (165-327) [17/18]	0.749
**Treatments**, n (%)			
NSAID/COX-ib	3 (5)	3 (17)	0.120
LMWH	25 (44)	14 (78)	**0.012**
SARS-CoV-2 antivirals	27 (47)	0 (0)	**<0.001**
Corticosteroids	23 (2)	12 (67)	0.051
Tocilizumab	1 (2)	1 (6)	0.383
**Outcome**, n (%)			
Hospital admission	32 (56)	17 (94)	**0.003**
Transfer to ICU	3 (9)	3 (18)	0.120
Discharge	29 (91)	13 (76)	0.112
Death	0 (0)	1 (6)	0.073
Time admission to outcome (days), median (IQR)	11 (5-25)	14 (10-23)	0.849
30-day mortality	0 (0)	3 (17)	**0.001**
**Complications**, n (%)			
Bleeding events	1 (2)	1 (6)	0.383
Thrombotic events	0 (0)	1 (6)	0.073

**Legend**. COV_δ-norm_, normal δ-granule content; COV_δ-low_, low δ-granule content; IQR, interquartile range; ADP, adenosine diphosphate; ATP, adenosine triphosphate; WBC, white blood cell; NL ratio, Neutrophil-Lymphocyte Ratio; PF-ratio, PaO_2_/FIO_2_ ratio; NSAID, non-steroidal anti-inflammatory drugs; COX-ib, COX-2 inhibitor; LMWH, Low-molecular-weight heparin; ICU, Intensive Care Unit. Statistical analyses: Mann-Whitney test, unpaired *t*-test, Fisher's exact test, or Chi-square test, as appropriate. Two-tailed *P*-values <0.05 were deemed statistically significant (in bold).

### SARS-CoV-2 RNAemia and Platelet Degranulation

**Figure 1.**
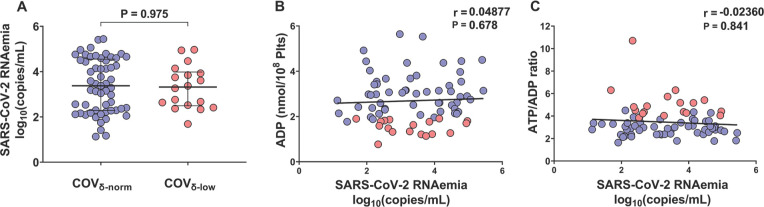
Plasmatic SARS-CoV-2 RNAemia in patients with COVID-19 according to their platelet δ-granule content. (A) Plasmatic SARS-CoV-2 RNAemia in patients with COVID-19 patients with normal platelet δ-granule content (COV_δ-norm_, n=57) and low platelet δ-granule content (COV_δ-low_, n=18). (B) Correlations between SARS-CoV-2 RNAemia log_10_(copies/mL) with platelet ADP content (nmol/10^8^ platelets) and (C) platelet ATP/ADP ratio. Median values with interquartile ranges (IQR) are shown for each group of patients. Data were analyzed using the Mann-Whitney test and the Spearman's correlation test. Statistical significance was assumed for two-sided *P*-values <0.05.

Firstly, we assessed SARS-CoV-2 RNAemia in the study population. There were no statistically significant differences in SARS-CoV-2 RNAemia between the COV_δ-norm_ and COV_δ-low_ groups [3.4 median log_10_(copies/mL), IQR 2.3 to 4.6 *vs* 3.3 median log_10_(copies/mL), IQR 2.5 to 4.0, respectively, *P*=0.9755] (Figure 1A). Furthermore, no statistically significant correlations were observed between the markers used to assess platelet degranulation and SARS-CoV-2 RNAemia, neither when considering the entire cohort (ADP: r=0.05, *P*=0.678; ATP/ADP ratio: r=-0.024, *P*=0.841) (Figure 1B-C) nor when patients were stratified based on their platelet degranulation status (COV_δ-norm_: ADP content r=0.078, *P*=0.565; ATP/ADP ratio: r=-0.025, *P*=0.856; COV_δ-low_: ADP content r=-0.046, *P*=0.855; ATP/ADP ratio: r=-0.168, *P*=0.504). These findings do not support an association of SARS-CoV-2 RNAemia with platelet degranulation.

### Peripheral Inflammation and Platelet Degranulation

**Figure 2.**
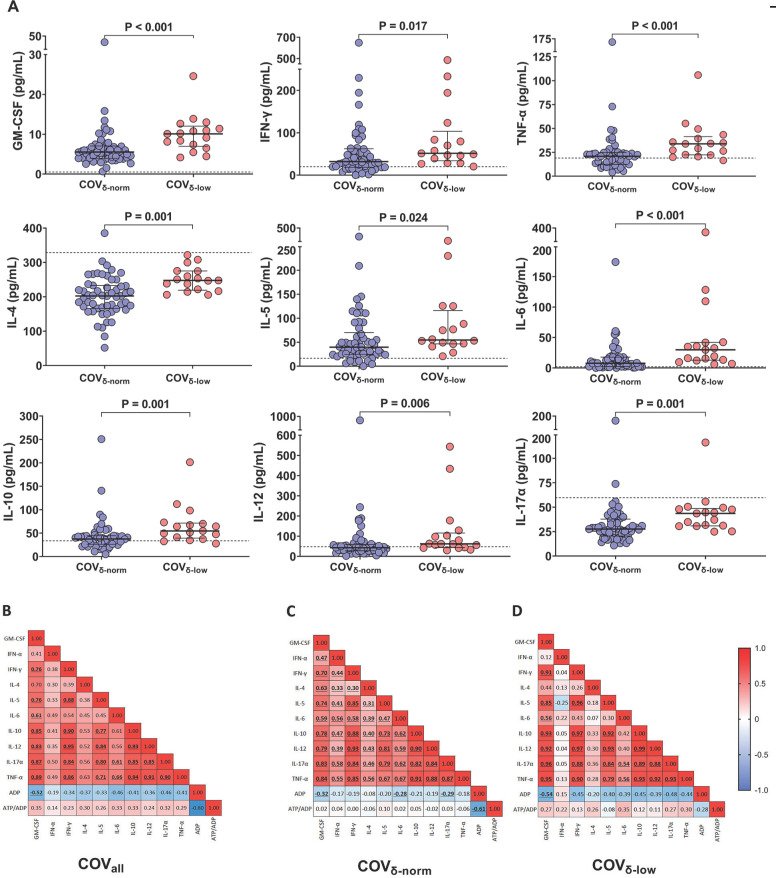
Plasmatic chemokine and cytokine levels in patients with COVID-19 according to their platelet δ-granule content. (A) Plasmatic chemokine and cytokine levels in patients with COVID-19 with normal platelet δ-granule content (COV_δ-norm_) and low platelet δ-granule content (COV_δ-low_). GM-CSF (n=55 and n=17), IFN-γ (n=53 and n=17), TNF-α (n=54 and n=17), IL-4 (n=55 and n=17), IL-5 (n=54 and n=16), IL-6 (n=53 and n=17), IL-10 (n=52 and n=17), IL-12 (n=53 and n=17), IL-17A (n=55 and n=17). Dots represent individual values. For each patient group, median values with interquartile ranges (IQR) are displayed. The dashed lines represent the median cytokine and chemokine value of 7 healthy subjects sampled before the pandemic. (B) Heatmap showing correlations between plasmatic chemokine and cytokine levels and platelet δ-granule content across the entire cohort of patients with COVID-19, (C) only in COV_δ-norm_ patients, and (D) only in COV_δ-low_ patients. The color gradient represents the correlation coefficient (r); numerical values within each cell indicate the r value. Statistically significant correlations are highlighted in bold and underlined. Data were analyzed using the Mann–Whitney or *t*-test, as appropriate, and the Spearman's correlation test. Statistical significance has been assumed for two-sided *P*-values <0.05.

Next, we evaluated the peripheral cytokine milieu in both patient groups. Significantly higher levels of GM-CSF, IFN-γ, TNF-α, IL-4, IL-5, IL-6, IL-10, IL-12, and IL-17A were observed in the COV_δ-low_ group compared to the COV_δ-norm_ group (Figure 2A), with the exception of IL-2 and IL-9, which were detectable only in 3 patients (data not shown). Median cytokine and chemokine values of 7 healthy participants sampled before the pandemic are included as reference (Figure 2A).

Since ADP is stored in platelet dense granules and released upon activation, intracellular ADP levels drop following degranulation. Meanwhile, ATP levels remain relatively stable, resulting in an increased ATP/ADP ratio. When evaluating the correlation between plasma cytokine and chemokine levels and ADP content across the entire patient cohort, a trend towards a negative correlation was observed, reaching statistical significance for GM-CSF (r=-0.52, *P*<0.001) (Figure 2B). Similar patterns were noted when stratifying patients based on their platelet degranulation status. The correlation between ADP and GM-CSF remained significant in both groups (COV_δ-norm_: r=-0.32, *P*=0.02; COV_δ-low_: r=-0.54, *P*=0.03) (Figure 2C-D). Statistical significance was also achieved, in the COV_δ-norm_ group only, for IL-6 (r=-0.28, *P*=0.04) and IL-17α (COV_δ-norm_: r=-0.29, *P*=0.03) (Figure 2C). The positive correlation trend that we observed between plasma cytokine and chemokine levels with the ATP/ADP ratio in the overall cohort appeared to be mainly driven by the COV_δ-low_ group (Figure 2D). Negative correlations between intracellular ADP levels and plasma cytokine concentrations, along with positive correlations between the ATP/ADP ratio and the same cytokines, indicate that greater platelet degranulation, reflected by lower ADP content and a higher ATP/ADP ratio, is associated with increased systemic inflammation. These findings suggest that plasma cytokine and chemokine levels are associated with platelet degranulation, with a stronger association in patients with more pronounced platelet degranulation. Indeed, whereas in the COV_δ-norm_ group, only ADP appears to be negatively correlated with inflammation; in the COV_δ-low_ group, both parameters used to assess platelet degranulation appear to be associated with inflammation. Specifically, ADP was negatively correlated with inflammation, and as expected, ATP/ADP positively correlated with inflammation.

**Figure 3.**
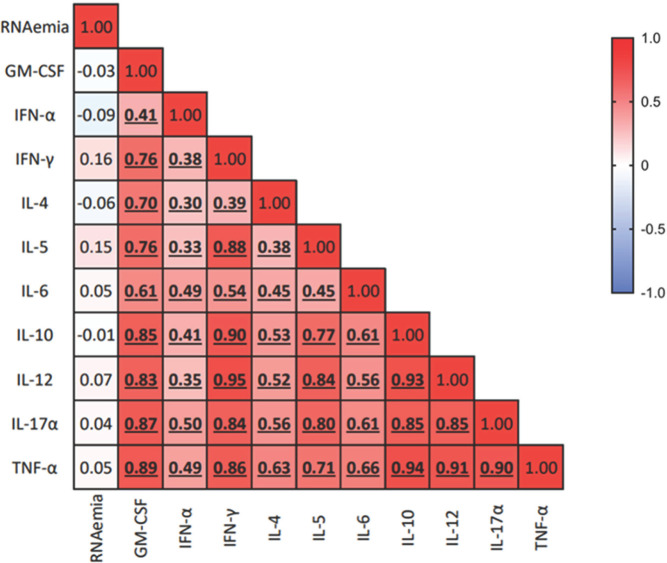
Association of SARS-CoV-2 RNAemia with the peripheral cytokine milieu. Heatmap showing correlations between SARS-CoV-2 RNAemia log_10_(copies/mL) and plasmatic chemokine and cytokine levels (pg/mL). Spearman's correlation test was used to assess correlation between variables; the color gradient represents the correlation coefficient (r); numerical values within each cell indicate the r value. *P*-values <0.05 are considered statistically significant, and statistically significant correlations are highlighted in bold and underlined.

Notably, whereas platelet degranulation appears to be associated with peripheral inflammation, in this group of individuals, SARS-CoV-2 RNAemia does not seem to be associated with inflammation, suggesting the involvement of other mechanisms (Figure 3).

### Association of Platelet Activation with SARS-CoV-2 RNAemia and the Peripheral Cytokine Milieu

Platelet activation and degranulation are distinct yet interconnected functional phases. Although activation typically precedes degranulation, it does not inevitably lead to it; platelets can become activated without releasing their granular contents. Given this functional dissociation, it is important to assess both processes independently. Having evaluated platelet degranulation and its association with systemic inflammation, we next focused on platelet activation per se to further explore its potential relationship with SARS-CoV-2 RNAemia and the peripheral cytokine milieu.

**Figure 4.**
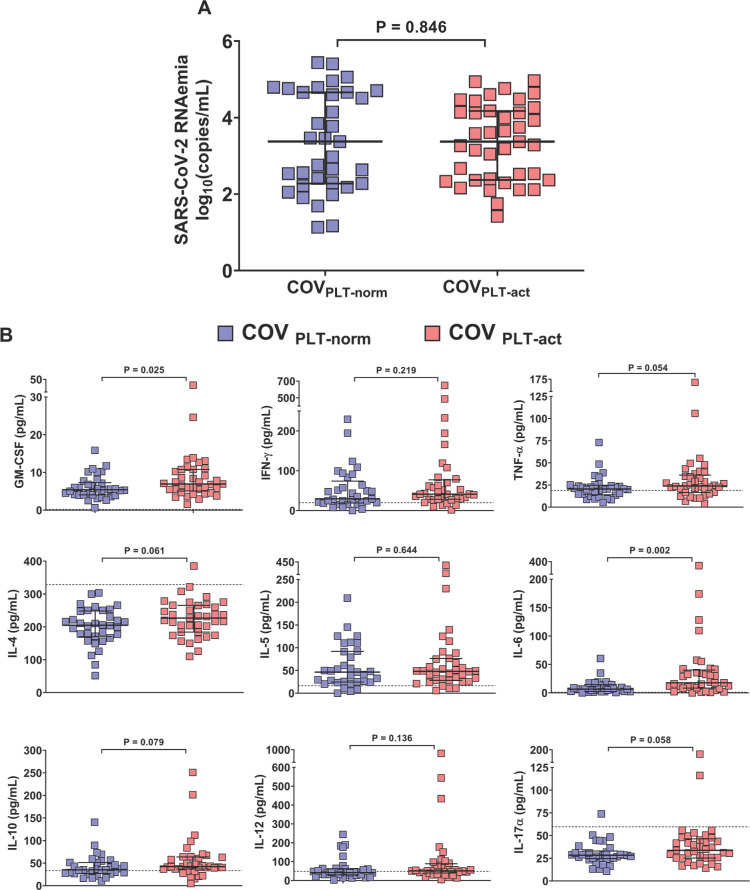
Association of platelet activation with SARS-CoV-2 RNAemia and the peripheral cytokine milieu. Patients with COVID-19 were categorized into two groups according to the cut-off for PMAs measured in 36 healthy subjects: COV_PLT-norm_ (n=39) had PMA levels below the cut-off of 23.02%, whereas COV_PLT-act_ (n= 35) had PMA levels above the cut-off. (A) Plasmatic SARS-CoV-2 RNAemia in patients with COVID-19 without circulating activated platelets (COV_PLT-norm_, n=35) and with circulating activated platelets (COV_PLT-act_, n=39) [COV_PLT-norm_: 3.4 median log_10_(copies/mL), IQR 1.4-4.2, vs COV_PLT-act_: 3.4 median log_10_(copies/mL), IQR 2.3-4.7, respectively, p=0.846]. (B) Plasmatic chemokine and cytokine levels in COVID-19 patients without circulating activated platelets (COV_PLT-norm_) and with circulating activated platelets (COV_PLT-act_). GM-CSF (n=34 and n=37), IFN-γ (n=33 and n=36), TNF-α (n=33 and n=37), IL-4 (n=34 and n=37), IL-5 (n=33 and n=36), IL-6 (n=33 and n=36), IL-10 (n=32 and n=36), IL-12 (n=33 and n=36), IL-17A (n=34 and n=37). Median values with interquartile ranges (IQR) are shown for each group of patients. Data were analyzed using the Mann–Whitney or *t*-test, as appropriate, and the Spearman's correlation test. Statistical significance has been assumed for two-sided *P*-values <0.05.

To investigate the relationship between platelet activation, SARS-CoV-2 RNAemia, and systemic inflammation, we stratified patients with COVID-19 into 2 groups based on PMAs. The cut-off for PMAs (23.02%) was derived from measurements in 36 healthy donors. Patients with PMA levels below this threshold were classified as COV_PLT-norm_ (n=39), whereas those with PMA levels above the threshold were designated as COV_PLT-act_ (n=35). SARS-CoV-2 RNAemia did not differ significantly between the 2 groups (COV_PLT-norm_: median 3.4 log_10_[copies/mL], IQR 1.4–4.2; COV_PLT-act_: median 3.4 log_10_[copies/mL], IQR 2.3–4.7; p=0.846) (Figure 4A).

Plasma cytokine and chemokine profiling revealed significantly higher concentrations of GM-CSF and IL-6 in the COV_PLT-act_ group compared to COV_PLT-norm_ (*P*=0.027 and *P*=0.002, respectively). Other cytokines, including TNF-α, IL-4, IL-10, and IL-17α, were modestly elevated in the COV_PLT-act_ group but did not reach statistical significance. Similarly, IFN-γ, IL-5, and IL-12 levels did not differ significantly between the 2 groups (Figure 4B). Therefore, these data may suggest that peripheral inflammation is more strongly associated with degranulation than with activation, most likely due to the intrinsic functional significance of degranulation.

## DISCUSSION

In this study, we investigated the complex interplay between SARS-CoV-2 RNAemia and systemic inflammation in the onset of platelet dysfunction in a cohort of patients with COVID-19 during the acute phase of the disease. We and others have described the presence of circulating degranulated platelets in patients with COVID-19 [9, 21] and wondered if their presence might be a hallmark of the alterations of the haemostatic system leading to both thrombosis and bleeding in these patients. The mechanisms leading to the detection of circulating degranulated platelets during acute SARS-CoV-2 infection, however, remain obscure. According to our findings, although SARS-CoV-2 RNAemia was detectable in the blood of patients with COVID-19, it was not associated with platelet dysfunction or platelet activation. We have found that plasma levels of cytokines and chemokines were strongly associated with the investigated parameters of platelet dysfunction. Thus, our results suggest that peripheral inflammation is more strongly associated with platelet dysfunction than the levels of SARS-CoV-2 RNAemia.

The detection of SARS-CoV-2 RNAemia has been a topic of considerable interest, as it may reflect viral dissemination beyond the respiratory tract [33] and has been associated with poor clinical outcomes and immune dysfunction [14, 31, 34]. In our cohort, SARS-CoV-2 RNAemia was detected across a wide range of patients with varying disease severity (ie, both outpatients and hospitalized). However, RNAemia did not show a statistically significant correlation with platelet degranulation. Therefore, the presence of viral RNA in the bloodstream alone may not be directly linked to the platelet dysfunction observed in patients with COVID-19. However, it is plausible that SARS-CoV-2 RNAemia might affect platelet function through other pathways, such as endothelial damage or immune activation, which were not investigated in this study.

In contrast, we found that pro-inflammatory cytokines and chemokines were significantly elevated in patients with circulating degranulated platelets, with strong correlations between the levels of IL-6 and GM-CSF and parameters of platelet dysfunction. These findings point towards a crucial role of peripheral inflammation in platelet dysfunction of COVID-19, with cytokines possibly acting in synergy with mediators like ADP or thromboxane A2 [35–37] to exacerbate the prothrombotic state. Although platelets do not directly produce the cytokine IL-6, which was measured in our study, they can still indirectly contribute to its systemic levels. Notably, activated platelets can release IL-1β, a potent pro-inflammatory cytokine capable of stimulating endothelial cells, monocytes [38], and other immune cells to produce secondary cytokines, including IL-6. Therefore, while platelets are not a direct source of IL-6, they may contribute to its upregulation via the release of upstream mediators.

The clinical implications of these findings are significant. Platelet dysfunction has been associated with thrombotic [6, 39–43] and bleeding complications [5] in severe COVID-19. Our findings suggest the potential benefit of targeting inflammation to prevent or mitigate platelet-related complications in COVID-19.

Our study has limitations that should be acknowledged. Firstly, the sample size, and particularly that of the COV_δ-low_ subgroup, was relatively small. Additionally, we did not directly quantify SARS-CoV-2 RNA within platelets, thus our study does not contribute to the unresolved question of whether platelets are directly infected by the virus. It is also important to note that our study excluded patients on antiplatelet treatment, so our findings cannot be extended to this population and do not clarify whether pre-existing antiplatelet therapy alleviates or exacerbates platelet dysfunction in COVID-19 patients. Finally, although we focused on the presence of circulating degranulated and activated platelets, we acknowledge that many other contributors (eg, endothelial dysfunction, von Willebrand factor activity, thrombin generation) play a role in the onset of thrombotic and bleeding complications in patients with COVID-19.

In conclusion, our study provides new insights into the pathophysiology of platelet dysfunction in COVID-19, emphasizing the pivotal role of inflammation, rather than RNAemia, in driving these changes. Our findings suggest that early therapeutic strategies aimed at controlling inflammation may be crucial for the prevention of the thrombotic and bleeding complications seen in severe COVID-19. Further research is needed to deepen our understanding of the mechanisms linking inflammation to platelet dysfunction in COVID-19 as well as in other viral and bacterial infections, with the final aim of developing targeted interventions that could improve patient outcomes.
